# A favorable outcome of dengue hemorrhagic fever despite poor prognostic indices: a case report with a mix of classic and unusual clinical and laboratory features

**DOI:** 10.11604/pamj.2019.34.74.20373

**Published:** 2019-10-05

**Authors:** Olukemi Adekanmbi, Sulaiman Lakoh

**Affiliations:** 1 Department of Medicine, University of Ibadan, Ibadan, Nigeria; 2 Department of Medicine, University College Hospital, Ibadan, Nigeria; 3 Department of Medicine, University of Sierra Leone, Freetown, Sierra Leone; 4 Department of Medicine, University of Sierra Leone Teaching Hospitals Complex, Freetown, Sierra Leone

**Keywords:** Dengue, fever, viral hemorrhagic fever

## Abstract

The report describes a 32-year-old man with dengue hemorrhagic fever presenting with acute onset high-grade intermittent fever with chills and rigors, headache, myalgia, abdominal pain, and vomiting. His laboratory results revealed neutrophilia, thrombocytopenia, microscopic hematuria, and a markedly elevated D-dimer. While on admission, he developed diarrhea, hypertension, and respiratory symptoms which evolved into respiratory distress with low oxygen saturation, eventually warranting his admission to the Intensive Care Unit (ICU). Despite his adverse prognostic indices, the patient made an uneventful recovery with conservative management after 16 days of admission. Thus illustrating how aggressive management could influence the outcome of dengue illness.

## Introduction

Dengue fever is a viral infection with anthroponotic transmission through the bite of an infected mosquito, most often *Aedes aegypti* [1]. The World Health Organization (WHO) defined dengue as an acute febrile illness, with two or more of the following signs or symptoms: an intense headache, retro-orbital pain, myalgia, arthralgia, skin rash, leucopenia and hemorrhagic manifestations [2]. Despite being an emerging infectious disease in Nigeria, interventions for the prevention and control of dengue are limited [3]. Complicating matters is the lack of surveillance and few diagnostic facilities resulting in the frequent misdiagnosis of dengue illness as malaria [3]. When severe, dengue infections may carry a high mortality [4]. Low platelet count and elevated D-dimers are among the reported indicators of severe dengue fever designated as dengue hemorrhagic fever and dengue shock syndrome [5]. According to WHO, dengue hemorrhagic fever is the presence of fever, or a recent history of fever lasting 2-7 days, any hemorrhagic manifestation, thrombocytopenia (platelet count of <100,000/μl) and evidence of increased vascular permeability [2]. Reports describing favorable response to treatment of patients with dengue in the setting of poor prognostic indices is limited. In this case report, we describe a case of dengue hemorrhagic fever with a marked elevation of D-dimer and thrombocytopenia that was successfully managed with conservative care.

## Patient and observation

A 32-year-old man with no antecedent comorbidities presented to the emergency department of a tertiary hospital in South Western Nigeria with an acute onset high-grade intermittent fever with chills and rigors, headache, myalgia, abdominal pain, and vomiting. There was no reported diarrhea or yellowness of the eyes. Prior to presentation, he had used oral anti-malaria medications, oral ciprofloxacin and antipyretics with no improvement. He worked as an agricultural research fellow and routinely spent a fair amount of time at work outdoors in farms and fields. Physical examination showed an acutely ill looking febrile patient with a temperature of 38.9°C, blood pressure of 120/70mmHg, a pulse of 92 beats per minute, and a respiratory rate of 24 cycles per minute. The rest of his physical examination was normal. His initial laboratory evaluation showed a leukocyte count of 7,090/μl of which 84.7% were neutrophils, a hemoglobin of 14.5g/dl, and platelet count of 89,000/μl. Activated Partial Thromboplastin Time (APTT) and prothrombin time were prolonged by 15.2 and 5.3 seconds, respectively. A slightly elevated INR of 1.35, but a markedly elevated D-dimer of 224.6μg/ml (ref 0-0.5μg/ml) were observed. The renal biochemical parameters were normal except for creatinine of 1.5mg/dl. Apart from an AST of 223 IU/l and an ALT of 70IU/l, all other liver function tests were normal. Urinalysis revealed microscopic hematuria. Other investigations are detailed in Table 1. Intravenous ciprofloxacin, metronidazole, and parenteral artesunate were commenced.

**Table 1:** Results of laboratory investigations

Laboratory variable	Reference range/control (Adult)	On admission Day 2	On admission Day 4	On admission Day 6	On admission Day 7	On admission Day 11
**Full Blood Count**						
Hemoglobin (g/dl)	12.0-16.0	14.5		14.2		11.9
Hematocrit (%)	36.0-49.0	41.9		41.2		35.8
White cell count (/μl)	4000-11000	7090		11720		8820
Neutrophils (%)	37-72	84.7		66.6		55.6
Lymphocytes (%)	20-40	11.4		24.9		28
Platelet (/μl)	150-400,000	89,000		76,000		220,000
**Electrolytes, Urea and Creatinine**						
Na^+^ (mmol/l)	130-145	139			140	
Cl^-^ (mmol/l)	95-110	104			105	
K^+^ (mmol/l)	3.5-4.5	3.7			3.8	
HCO_3_(mmol/l)	20-30	25			21	
Urea (mg/dl)	15-45	33			22	
Creatinine (mg/dl)	0.7-1.2	1.5			1.3	
**Urine**						
Protein	Negative	+	++	++		
Blood	Negative	++	++	+++		
Ketones	Negative	Trace	++	+		
Bilirubin	Negative	Negative	Negative	+++		
Glucose	Negative	Negative	+	+		
**Liver function test**						
Tot. Bilirubin (mg/dl)	0.2-1.0	0.4				
Dir. Bilirubin (mg/dl)	0.0-0.4	0.1				
AST (IU/L)	0-37	223				
ALT (IU/L)	0-40	76				
ALP (IU/L)	40-130	56				
GGT (IU/L)	7-50	45				
**Clotting and fibrinolysis**						
PT (s)	15.5				20.8	
APTT (s)	35				50.20	
INR	0.9-1.2				1.35	
D-dimer (μg/ml)	0.00-0.50				224.60	

On subsequent review, the temperature was 39.2°C, pulse was 102 beats per minute, and there was tenderness on the right iliac fossa and suprapubic areas. Ciprofloxacin was changed to ceftriaxone, and parenteral metoclopramide was added to control the vomiting which had persisted since admission. On the fourth day of admission, he complained of rhinorrhea. Further physical examination revealed a mildly inflamed pharynx with no significant regional lymph node enlargement. Pulse was 82 beats per minute, blood pressure 110/70mmHg, respiratory rate of 32 cycles per minute, and reduced breath sounds in the middle and lower zones. Chest X-ray showed perihilar patchy opacities worse on the right with few patchy opacities on the right upper and lower zones. With concern for a sub-optimally treated community-acquired pneumonia, azithromycin was added for atypical pathogens. The following day, the temperature was noticed to be 40.5°C despite regular antibiotics. Pulse was 100 per minute, blood pressure was 130-170/60-100mmHg. The respiratory rate was 62 cycles per minute, and SpO_2_ was 79-81% while breathing ambient air. Percussion notes were dull on the right lower zone, and breath sounds were vesicular with a mix of coarse crackles and rhonchi on the right lower zone.

A repeat laboratory evaluation showed worsening microscopic hematuria, thrombocytopenia, and a leukocytosis of 11,720/μl. Blood film for malaria parasite detection was repeatedly negative. Supplemental intranasal oxygen at 4L per minute was administered. Loratadine and amlodipine were added, and the patient was transferred to the Intensive Care Unit (ICU) for respiratory support. In the ICU, the dyspnea improved with oxygen therapy by face mask. SpO_2_ on oxygen therapy ranged between 93% and 97%. Respiratory rate was 26 cycles per minute, pulse was 88 beats per minute, and blood pressure was 135/76mmHg. However, he continued to have high-grade fever, tachypnea and developed diarrhea with dark-colored stools over the next 3 days while in the ICU. On the 10th day of admission, a positive result for viral hemorrhagic fever screening using real-time RNA polymerase chain reaction panel detecting the dengue virus was obtained. Supportive management was continued. After 16 days of conservative management, the patient had an uneventful recovery and was discharged home from the hospital. He was established to have a normal blood pressure with no medication after a year of follow up.

## Discussion

We described a good outcome of dengue illness with a mix of classical and unusual clinical and laboratory features in the setting of poor prognostic indices (marked elevation of D-dimer, deranged clotting profile and low platelet count [4, 5]. Dengue fever is an acute febrile clinical syndrome associated with headache, retro-orbital pain, myalgia, arthralgia, skin rash, leucopenia and hemorrhagic manifestations [2]. Although these symptoms are in keeping with those experienced by the patient described in this case report, many of them are nonspecific and could be found in a number of common infections, making an early diagnosis difficult. In Nigeria and other countries with high malaria endemicity, patients with this presentation are often misdiagnosed and treated as malaria [3]. This was the practice in the case described above even with repeatedly negative malaria tests. Accordingly, a high index of suspicion and availability of quick turn-around diagnostic services are critical to early diagnosis and management of dengue hemorrhagic fever and indeed other viral hemorrhagic fevers.

Over the course of his admission, the patient developed diarrhea and respiratory symptoms though these are unusual manifestations of dengue illness [4]. In contrast to the usual manifestations of hypotension in severe dengue [2], the patient also developed hypertension while on admission that was severe enough to warrant treatment. Nonetheless, normal blood pressures without treatment were established after a year of follow up. In a retrospective review of 385 dengue cases in Thailand, 26.2% had hypertension [6]. Thus, using low blood pressure as a severity predictor in dengue illness might not be reliable. Evidence from the literature has highlighted comorbidities like hypertension in dengue illness as severity indicators [7] that require aggressive but diligent management. The initial neutrophilia and subsequent leukocytosis in this patient are unusual as the typical picture in dengue is a low white cell count of ≤5000/μl [4].

Our patient received multiple antibiotic regimens. Although empiric antibiotics are indicated in the management of other viral hemorrhagic fevers with severe illness [8], they are not routinely recommended in patients with dengue illness [9]. Nonetheless, delays in the detection of dengue illness may potentially increase the chance of antibiotic overuse. As dengue remains a major public health threat in low resource settings, it is critical that healthcare workers improve their understanding of the diagnosis and management of the disease. Predictors of poor outcomes in a patient with dengue illness are elevated levels of D-dimer and thrombocytopenia [5]. Our patient had a platelet count of 89,000/μl which is below the threshold (≤100,000/μl) for a signal of plasma leakage in dengue hemorrhagic fever [4]. Mortality in dengue illness is higher when the condition progresses to dengue hemorrhagic fever [4]. D-dimer was over 400 times above the upper limit of the laboratory reference range. Even in the face of these severity indicators, the patient was discharged with no complication or comorbidity at 1 year follow up.

## Conclusion

We have described a case of dengue hemorrhagic fever with several predictors of severity, but a good outcome after presenting with both classic and unusual clinical and laboratory features. A good understanding of the case definition of dengue fever by healthcare workers in LMICs and widening the armamentarium for establishing various diagnoses of febrile illness to include dengue fever are critical steps to improve detection and management of dengue illness. Clinicians should closely monitor patients with dengue illness for features of severity in order to avert mortality among these patients. Rapid diagnostic tests for dengue virus should be made available to health facilities in low resource settings for early detection of dengue illness. Community awareness programs are crucial to the prevention and control of dengue hemorrhagic fever.

## Competing interests

The authors declare no competing interests.
